# Analysis of β-lactamase phenotypes and carriage of selected β-lactamase genes among *Escherichia coli* strains obtained from Kenyan patients during an 18-year period

**DOI:** 10.1186/1471-2180-12-155

**Published:** 2012-07-28

**Authors:** John Kiiru, Samuel Kariuki, Bruno M Goddeeris, Patrick Butaye

**Affiliations:** 1 Centre for Microbiology Research, Kenya Medical Research Institute, P.O Box 19464-00202, Nairobi, Kenya; 2 Department of Biosystems, Faculty of Bio-Science Engineering, Katholieke Universiteit Leuven, Kasteelpark Arenberg 30, B-3001, Heverlee, Belgium; 3 Veterinary and Agrochemical Research Centre, Groeselenberg 99, B-1180, Ukkel, Belgium; 4 Department of Virology, Parasitology and Immunology, Faculty of Veterinary Medicine, University of Ghent, Salisburylaan 133, 9820, Merelbeke, Belgium; 5 Department of Pathology, Bacteriology and Poultry Diseases, Faculty of Veterinary Medicine, University of Ghent, Salisburylaan 133, 9820, Merelbeke, Belgium

## Abstract

**Background:**

Although β-lactam antibiotics are heavily used in many developing countries, the diversity of β-lactamase genes (*bla*) is poorly understood. We screened for major β-lactamase phenotypes and diversity of *bla* genes among 912 *E. coli* strains isolated from clinical samples obtained between 1992 and 2010 from hospitalized and non-hospitalized patients.

**Results:**

None of the isolates was resistant to carbapenems but 30% of all isolates were susceptible to cefepime, cephamycins and piperacillin-tazobactam. Narrow spectrum β-lactamase (NSBL) phenotype was observed in 278 (30%) isolates that contained *bla*_*TEM*-1_ (54%) or *bla*_*SHV*-1_ (35%) or both (11%). Extended Spectrum β-lactamase (ESBL) phenotype was detected in 247 (27%) isolates which carried *bla*_*CTX-M*-14_ (29%), *bla*_*CTX-M*-15_ (24%), *bla*_*CTX-M*-9_ (2%), *bla*_*CTX-M*-8_ (4%), *bla*_*CTX-M*-3_ (11%), *bla*_*CTX-M*-1_ (6%), *bla*_*SHV*-5_ (3%), *bla*_*SHV*-12_ (5%), and *bla*_*TEM-52*_ (16%). Complex Mutant TEM-like (CMT) phenotype was detected in 220 (24%) isolates which carried *bla*_*TEM*-125_ (29%), while *bla*_*TEM*-50_, *bla*_*TEM*-78_, *bla*_*TEM*-109_, *bla*_*TEM −*152_ and *bla*_*TEM*-158_ were detected in lower frequencies of between 7% and 11%. Majority of isolates producing a combination of CTX-M-15 + OXA-1 + TEM-1 exhibited resistance phenotypes barely indistinguishable from those of CMT-producers. Although 73 (8%) isolates exhibited Inhibitor Resistant TEM-like (IRT) phenotype, *bla*_*TEM*-103_ was the only true IRT-encoding gene identified in 18 (25%) of strains with this phenotype while the rest produced a combination of TEM-1 + OXA-1. The pAmpCs-like phenotype was observed in 94 (10%) isolates of which 77 (82%) carried *bla*_*CMY*-2_ while 18% contained *bla*_*CMY*-1._

Isolates from urine accounted for 53%, 53%, 74% and 72% of strains exhibiting complex phenotypes such as IRT, ESBL, CMT or pAmpC respectively. On the contrary, 55% isolates from stool exhibited the relatively more susceptible NSBL-like phenotype. All the phenotypes, and majority of the *bla* genes, were detected both in isolates from hospitalized and non-hospitalized patients but complex phenotypes were particularly common among strains obtained between 2000 and 2010 from urine of hospitalized patients.

**Conclusions:**

The phenotypes and diversity of *bla* genes in *E. coli* strains implicated in clinical infections in non-hospitalized and hospitalized patients in Kenya is worryingly high. In order to preserve the efficacy of β-lactam antibiotics, culture and susceptibility data should guide therapy and surveillance studies for β-lactamase-producers in developing countries should be launched.

## Background

β-lactam antibiotics are an important arsenal of agents used against both Gram-negative and Gram-positive bacteria. Resistance to this class of antimicrobials is therefore of immense clinical significance. It is important to investigate the epidemiology of strains that are resistant to β-lactam antibiotics especially in Sub-Saharan Africa where treatment with alternative or more effective agents may be beyond the reach of majority of patients. Before treatment using β-lactam antibiotics is initiated, proper and timely identification of the β-lactamase phenotype is of critical importance. Failure or delay to do this may lead to therapeutic failure and death of patients [1]. In order to guide therapy and in order to understand the molecular epidemiology of β-lactamase-producers, a combination of susceptibility profiling, PCR and sequencing techniques may be required [2-4]. These techniques are not always available or affordable in resource-poor settings. Therefore, the prevalence of β-lactamases in developing countries is largely undetermined and the use of β-lactam antibiotics in such countries remains largely empiric.

Based on resistance to β-lactam/β-lactamase inhibitor antibiotics, bacteria strains may be conveniently categorized into various resistant phenotypes [5]. Strains exhibiting Narrow Spectrum β-lactamase Phenotypes (NSBLs) normally produce TEM-1 and/or SHV-1 enzymes that effectively degrade penicillins but are susceptible to other classes of β-lactams [6]. However, mutations on the promoter region of the gene encoding TEM-1 may result to over-production of these otherwise narrow-spectrum enzymes. This overproduction may in turn confer resistance to other classes of β-lactams besides penicillins [7-10]. Point mutations on these enzymes may also generate inhibitor resistant enzymes such as the Inhibitor Resistant TEMs (IRTs) that degrade penicillins but are not impeded by β-lactamase inhibitors such clavulanic acid or sulbactam [4,11]. Extended Spectrum β-Lactamases (ESBLs) may also be derived from TEM- and SHV-type enzymes. ESBLs exhibit a wide hydrolytic ability to different generations of cephalosporins but remain susceptible to β-lactamase inhibitors [12]. Complex Mutant TEMs (CMTs) are also derived from TEM-1 or TEM-2 and degrade most β-lactams but are susceptible to β-lactamase inhibitors including tazobactam. The CMTs are also susceptible to cephamycins and carbapenems [13]. Plasmid–encoded AmpC (pAmpC) such as CMYs mediate resistance to most classes of β-lactams except to fourth generation cephalosporins and carbapenems [14]. The β-lactamases with the worst clinical implications are those that degrade carbapenems, the most potent class of β-lactam antibiotics available today. Some carbapenemases such as the *Klebsiella pneumoniae* carbapenemases (KPC) degrade virtually all classes of β-lactams [15-17]. Some carbapenemases such as metallo-β-lactamases (MBLs) are however susceptible to aztreonam, a monobactam [18]. It is therefore clear that determination of β-lactamase phenotypes may not only aid the choice of agents to treat patients but may also guide the screening of *bla* genes and therefore save costs in surveillance studies. Understanding molecular epidemiology of *bla* gene is also important because majority of broad-spectrum resistant enzymes, especially the ESBLs and CMYs are encoded in conjugative plasmids that may be acquired across species barrier. Therefore, such genes have a high potential for spread via horizontal gene transfer mechanisms [19-22].

The phenotypic diversity of β-lactamase-producers in Kenya is poorly described and the diversity of *bla* genes has not been properly investigated [23-28]. The aim of the current study was to determine the β-lactamase phenotypes and carriage of *bla* genes of critical importance in *E. coli* obtained from blood, stool and urine obtained from hospitalised and non-hospitalised patients seeking treatment in Kenyan hospitals during an 18-year period (1992 to 2010).

## Results

### Phenotypic diversity of β-lactamase-producers

None of the 912 isolates tested in this study were resistant to carbapenems. Cefepime, (a fourth generation cephalosporin), cefoxitin (a cephamycin), and piperacillin-tazobactam (TZP), were effective against majority (60%) of these isolates. The NSBL-like phenotype was the most dominant phenotype in our collection and was observed in 278 (30%) of the 912 isolates compared to 73 (8%), 247 (27%), 220 (24%) and 94 (10%) of isolates found to exhibit IRT-, ESBL-, CMT and pAmpC-like phenotypes respectively, Table 1. Based on resistance phenotypes, 247 ESBL-producers fit into two sets. The first set comprised of 142 isolates exhibiting resistance to combinations of aztreonam and multiple cephalosporins including ceftazidime. The other set of 105 isolates were resistant to the same panel of antibiotics but not to ceftazidime. The 220 isolates with a CMT-like phenotype were resistant to all generations of cephalosporins but were susceptible to cephamycins and carbapenems. Resistance to all β-lactamase inhibitors including TZP was observed in 160 (73%) of the CMT-producers. Among 40 isolates with a CMT-like phenotype that had intermediate resistance to TZP, tiny ghost zones (≤ 3 mm) were observed between amoxicillin-clavulanic acid (AMC) and ceftazidime (CAZ) and/or Cefotaxime (CTX). These isolates therefore exhibited a combination of both ESBL- and CMT-like phenotypes. The most resistant strains were those exhibiting a pAmpC-like phenotype. These 94 isolates comprising about 10% of all the isolates in our collection were resistant to most generations of cephalosporins and β-lactamase inhibitors including TZP but were susceptible to carbapenems.

**Table 1:** β-lactamase phenotypes encountered among the 912 strains analyzed

**Antibiotics to which isolates were resistant**						
**Penicillins, 1st & 2nd generation cephalosporins**	**3rd Generation cephalosporins &** M**onobactams**	**4th Generation cephalosporins**	**inhibitors**	**Cephamycins**	**Most probable Phenotype**^**a**^	**Total (%)n = 912**
AMP, KF, AMX	−	−	−	−	NSBL	103 (11)
AMP, AMX, KF OXA	−	−	−	−	NSBL	175 (19)
AMP, AMX, KF OXA	−	−	AMC, AMS	−	IRT	65 (7)
AMP, KF, AMX,	−	−	AMC, AMS	−	IRT	8 (1)
AMP, AMX, KF, CXM	CTX^b^, AZT^b^	−	−	−	ESBL	105 (12)
AMP, AMX , KF, CXM	CTX, CAZ^*^, AZT	−	−	−	ESBL	75 (8)
AMP, AMX, OXA KF, CXM	CTX^b^, CAZ^b^, AZT	FEP	AMS	−	ESBL	67 (7)
AMP, AMX, OXA KF, CXM	CTX, CAZ^*^, AZT	FEP	AMC, AMS	−	CMT	40 (4)
AMP, AMX, OXA, KF, CXM	CTX, CAZ, AZT	FEP	AMC, AMS, TZP	−	CMT	180 (20)
AMP, AMX, OXA KF, CXM	CTX, CAZ, AZT	FEP	AMC, AMS, TZP	FOX	pAmpC	94 (10)

Resistance phenotypes of the 912 isolates investigated.

a: β-lactamase phenotypes observed in different isolates were defined as follow:- Narrow spectrum β-lactamases (NSBLs) were resistant to penicillins but were susceptible to other classes of β-lactam antibiotics. Isolates exhibiting the inhibitor resistant TEM phenotype (IRT) were those capable of degrading penicillins, were not inhibited by β-lactamase inhibitors but were susceptible to other classes of β-lactam antibiotics. The ESBL-producers were resistant to penicillins, 2^nd^ and most 3^rd^ generation cephalosporins, and exhibited intermediate resistance to 4^th^ generation cephalosporins and were fully susceptible to cephamycins, carbapenems and β-lactamase inhibitors. The complex mutant TEMs (CMTs) were resistant to most β-lactams and β-lactamase inhibitors including TZP but were susceptible to cephamycins and carbapenems. Isolates with the pAmpC phenotypes were resistant to all generations of β-lactam antibiotics, were susceptible to carbapenems and were either susceptible or exhibited intermediate resistance to 4^th^ generation cephalosporins.

^b^: appearance of zones of synergy between a given cephalosporin or monobactam and amoxicillin-clavulanic acid (AMC).

(−) isolate with a given phenotype were susceptible to a given set of antibiotics.

### Distribution of β-lactamase-producers

All the β-lactamase phenotypes reported in this study were observed in isolates from all specimen-types obtained during the 1990s and 2000s and from both hospitalized and non-hospitalized patients, Table 2. While majority of isolates from stool exhibited the relatively susceptible NSBL-like phenotype, isolates from urine accounted for 55%, 53%, 57% and 72% of strains with complex resistances such as IRT-, ESBL-, CMT- and pAmpC-like phenotypes respectively. Majority of isolates from hospitalized patients, especially those diagnosed with UTIs, exhibited such complex phenotypes compared to those obtained from patients seeking outpatient treatment. These complex resistances were also more common among isolates obtained in recent years (2000–2010).

**Table 2:** Clinical background of strains exhibiting different β-lactamase phenotypes

		**Specimen-type**			**Patient category**		**Year of isolation**	
	**Total**	**Stool**	**Urine**	**Blood**	**Inpatient**	**Outpatient**	**1990s**	**2000s**
NSBL	278	153 (55)	39 (14)	86 (31)	82 (29)	196 (71)	186 (67)	91 (33)
IRT	73	18 (25)	38 (53)	17 (22)	60 (82)	13 (18)	28 (38)	45 (62)
ESBL	247	65(26)	130 (53)	52 (21)	170 (69)	77 (31)	79 (32)	168 (68)
CMT	220	21 (10)	163 (74)	36 (16)	163 (74)	57 (26)	62 (28)	158 (72)
pAmpC	94	13 (14)	68 (72)	13 (14)	87 (92)	7 (8)	12 (13)	82 (87)

Number (%) of isolates exhibiting a given phenotype among those obtained from different specimen-types and different category of patients during the 1990s and 2000s period.

### Carriage of *bla* genes

Carriage of *bla*_*TEM-*1_ or *bla*_*SHV*-1_ was associated with the NSBL-like phenotype in 54% and 35% of the 155 isolates exhibiting this phenotype respectively. The two genes were also found together in 11% of the NSBL-producers, Table 3. The only IRT-encoding gene identified in this study was *bla*_*TEM-*103_ that was detected in 18 (25%) of the 73 isolates with an IRT-like phenotype. The other 55 (75%) of isolates with this phenotype carried a combination of *bla*_*TEM-*1+_*bla*_*OXA*-1_ genes. Majority (78%) of the 247 isolates with an ESBL-like phenotype tested positive for CTX-M-type ESBLs. While *bla*_*CTX-M-*14_ and *bla*_*CTX-M*-15_ were detected in 29% and 24% of these isolates respectively, *bla*_*CTX-M-*1_, *bla*_*CTX-M*-3_, *bla*_*CTX-M*-9_ and *bla*_*CTX-M-*8_ were detected in lower frequencies of 6%, 11%, 2% and 4% respectively, Table 3. Isolates which carried *bla*_*CTX-M-*1_ alone exhibited intermediate resistances to aztreonam and cefotaxime and were fully susceptible to ceftazidime. The *bla*_*TEM*-52_ that was detected in 22 (16%) of ESBL-producers was the only TEM-type ESBL identified in this study. The carriage and diversity of SHV-type ESBL genes was also low in which case, only *bla*_*SHV-*5_ and *bla*_*SHV-*12_ ESBL-encoding genes were detected in 3% and 5% of the ESBL-producers respectively. Resistance to ceftazidime among the ESBL-producers was attributed mainly to carriage of *bla*_*CTX-M*-15_ or a combination of *bla*_*CTX-Ms*_ *+ bla*_*OXA-*1_ *+ bla*_*TEM-*1_ genes. A significant proportion (39%) of isolates containing *bla*_*CTX-Ms*_ or *bla*_*SHV*_-type ESBLs in the absence of *bla*_*OXA-*1_ or *bla*_*TEM-*1_ were susceptible to ceftazidime.

**Table 3:** Combination of β-lactamases detected in 586 strains analyzed

	**NSBL**	**IRT**	**ESBL**	**CMT**	**pAmpC**
**β-lactamase genes**	**n = 155**	**n = 73**	**n = 140**	**n = 124**	**n = 94**
TEM-1	84 (54)	−	−	−	−
SHV-1	54 (35)	−	−	−	−
TEM-1 and OXA-1	−	55 (75)	−	−	−
TEM-1 + SHV-1	17 (11)	−	−	−	−
SHV-5	−	−	4 (3)	−	−
SHV-12	−	−	7 (5)	−	−
CTX-M-1 + OXA-1	−	−	9 (6)	−	−
CTX-M-3	−	−	15 (11)	−	−
CTX-M-8	−	−	6 (4)	−	−
CTX-M-9	−	−	3 (2)	−	−
CTX-M-14	−	−	41 (29)	−	−
CTX-M-14 + TEM-1 + OXA-1	−	−	−	9 (7)	−
CTX-M-15	−	−	34 (24)	−	−
CTX-M-15 + TEM-1 + OXA-1	−	−	−	14 (11)	−
TEM-103	−	18 (25)	−	−	−
TEM-109	−	−	−	9 (7)	−
TEM-50	−	−	−	10 (8)	−
TEM-52	−	−	22 (16)	−	−
TEM-52 + OXA-1	−	−	−	15 (12)	−
TEM-78	−	−	−	9 (7)	−
TEM-125	−	−	−	36 (29)	−
TEM-152	−	−	−	14 (11)	−
TEM-158	−	−	−	10 (8)	−
CMY-1 + OXA-2	−	−	−	−	16 (17)
CMY-1	−	−	−	−	1 (1)
CMY-2	−	−	−	−	5 (5)
CMY-2 + SHV-5 + TEM-1	−	−	−	−	14 (15)
CMY-2 + SHV-12	−	−	−	−	12 (13)
CMY-2 + OXA-2	−	−	−	−	46 (49)

Combination of *bla* genes detected in isolates exhibiting different β-lactamase phenotypes.

(−) isolate with a given phenotype did not test positive for a given set of *bla* genes.

**Table 4:** Primers used for screening for β-lactamase genes

**Target Gene**	**Primer name**	**5'-3' sequence**	**T°C**	**Size (bp)**	**Gene accession number**
blaTEM	TEM-F	ATGAGTATTCAACAT TTC CG	55	840	EF125012-related
	TEM-R	CCAATGCTTAATCAG TGA GG			
blaSHV	SHV-F	TTCGCCTGTGTATTATCTCCCTG	50	854	AF148850-related
	SHV-R	TTAGCGTTGCCAGTGYTCG			
blaCTX-M concensus	MA1	ATGTGCAGYACCAGTAARGTKATGGC	60	593	Y10278-related
	MA2	TGGGTRAARTARGTSACCAGAAYCAGCGG			
CTX-M group I	CTXM1-F3	GAC GAT GTC ACT GGC TGA GC	55	499	X92506-related
	CTXM1-R2	AGC CG C CGA CGC TAA TAC A			
CTX-M group II	TOHO1-2 F	GCG ACC TGG TTA ACT ACA ATC C	55	351	X92507-related
	TOHO1-1R	CGG TAG TAT TGC CCT TAA GCC			
CTX-M group III	CTXM825F	CGC TTT GCC ATG TGC AGC ACC	55	307	AF189721-related
	CTXM825R	GCT CAG TAC GAT CGA GCC			
CTX-M group IV	CTXM914F	GCT GGA GAA AAG CAG CGG AG	62	474	AF252622-related
	CTXM914R	GTA AGC TGA CGC AAC GTC TG			
blaCMY (consensus)	CF1	ATGATGAAAAAATCGTTATGC	55	1200	U77414-related
	CF2	TTGCAGCTTTTCAAGAATGCGC			
blaCMY-1 group	CMY-1 F	GTGGTGGATGCCAGCATCC	58	915	AJ291609-related
	CMY-1R	GGTCGAGCCGGTCTTGTTGAA			
blaCMY-2 group	CMY-2 F	GCACTTAGCCACCTATACGGCAG	58	758	AF305559-related
	CMY-2R	GCTTTTCAAGAATGCGCCAGG			
blaOXA-1	OXA-1 F	ATGAAAAACACAATACATATCAACTTCGC	62	820	JO2967-related
	OXA-1R	GTGTGTTTAGAATGGTGATCGCATT			
blaOXA-2	OXA-2 F	ACGATAGTTGTGGCAGACGAAC	62	602	AF300985-related
	OXA-2R	ATYCTGTTTGGCGTATCRATATTC			
blaPER-concensus	PER-F	ATGAATGTCATTATAAAAGC	55	925	Z21957-related
	PER-R	AATTTGGGCTTAGGGCAGAA			
blaACC-like	ACC-F	AGCCTCAGCAGCCGGTTAC	53	818	AJ133121-related
	ACC-R	GAAGCCGTTAGTTGATCCGG			
blaVEB-concensus	VEB-F	ATTTAACCAGATAGGACTACA	55	1000	Z21957-related
	VEB-R	CGGTTTGGGCTATGGGCAG			
blaDHA-concensus	DHA-F	TGATGGCACAGCAGGATATTC	55	997	EF406115-related
	DHA-R	GCTTTGACTCTTTCGGTATTCG			

Primer combinations used for screening and sequencing *bla* genes. The consensus primers were used for screening and sequencing purposes except for *bla*_*CTX-M*_, and *bla*_*OXA*_ that were sequenced using group-specific primers. CTX-M group I primers detect genes encoding CTX-M −1, -3, -10 to −12, -15, -22,- 23, -28, -29 and −30 while primers for CTX-M group II primers detect genes encoding CTX-M-2, -4, -7, and −20. Group III primers detect only CTX-M-8 while group IV primers detect genes encoding CTX-M-9, -13, -14, -16 to 19, -21, and 27.

T°C: annealing temperature.

Y = T or C, R = G or A, S = G or C, K = G or T.

The *bla*_*TEM*-125_ was detected in 29% of the 124 isolates exhibiting a CMT-like phenotype and was therefore the most common CMT-encoding gene detected in this study. Other CMT genes: - *bla*_*TEM-50*_, *bla*_*TEM*-78_, *bla*_*TEM*-152_ and *bla*_*TEM*-158_ were detected in much lower prevalences of 8%, 7%, 11%, and 8% respectively, Table 3. Carriage of CMT genes did not account for CMT-like phenotypes in 30% of isolates with this phenotype. Nine of such isolates tested positive for a combination of *bla*_*TEM*-14_ + *bla*_*OXA*-1+_*bla*_*TEM*-1_ while 14 strains carried a combination of *bla*_*TEM*-15_ + *bla*_*OXA*-1+_*bla*_*TEM*-1._ Another 15 isolates tested positive for a combination of a *bla*_*TEM*-52_ (a TEM-type ESBL gene), and *bla*_*OXA-*1_. Production of OXA-1 and TEM-1 enzymes in the presence of CTX-M enzymes apparently masked the ESBL-phenotype that is otherwise conferred by CTX-M enzymes. Therefore, isolates producing a combination of such enzymes could hardly be distinguished from genuine CMT-producers. The *bla*_*CMY-*2_ that was present in 77 (72%) of all isolates in our collection was the most common pAmpC-encoding genes detected in this study. The CMYs were also detected in strains co-producing TEM-1 and SHV-type ESBLs suggesting a possible co-evolution of penicillinases, ESBLs and AmpCs genes in the same isolate. While majority of *bla*_*OXA*-1_ genes were detected in strains bearing ESBL genes such as *bla*_*CTX-Ms*_ or *bla*_*TEM-52,*_ the *bla*_*OXA*-2_ were detected in strains carrying *bla*_*CMYs*_ Table 3. None of the isolates investigated tested positive for *bla*-_*PER-*_like, *bla*_*ACC-*_like, *bla*_*VEB-*_like_*,*_ or *bla*_*DHA-*_like genes.

### Distribution of *bla* genes

**Figure 1:**
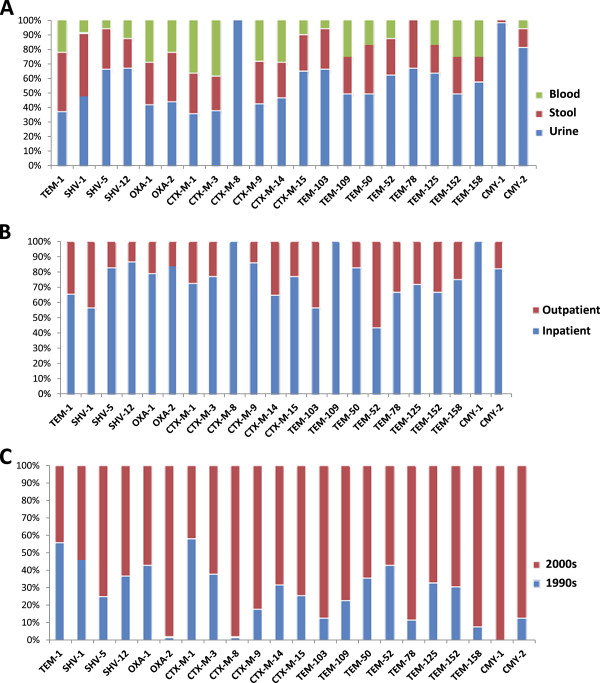
**Occurrence of *bla* genes among isolates from different clinical backgrounds.** 1**a**: Occurrence of *bla* genes among isolates from blood, stool and urine, 1**b**: Occurrence of *bla* genes among isolates from inpatient and outpatient populations: 1**c**: Occurrence of *bla* genes among isolates obtained in the 1990s and 2000s periods.

We also analyzed for the distribution of *bla* genes among strains obtained from different specimen-types and among those obtained from hospitalized and non-hospitalized patients, Figure 1. Majority of *bla* genes were present in all specimen-types regardless of their clinical backgrounds. However, *bla*_*CTX-M*-3_ was only detected in isolates from urine while *bla*_*TEM-*78_ was not detected among isolates from blood. *bla*_*TEM-109*_ and *bla*_*CTX-M*-8_ on the other hand_,_ were exclusively detected among isolates obtained from hospitalized patients. All *bla* genes described in this study were found in isolates obtained from both the 1990s and 2000s except *bla*_*CMY-*1_ that was exclusively detected among isolates obtained during the 2000–2010 period.

## Discussion

In this study, we describe the diversity of β-lactamase genes in a large collection of *E. coli* from different types of clinical specimen obtained from hospitalized and non-hospitalized patients in Kenya. This study suggests that carbapenems and to a less extent, cefepime, cephamycins and piperacillin-tazobactam may still be potent against majority of the isolates investigated. Although we do not rule out that the panel of *bla* genes in our strains is wider than what is reported in this study, there was a general agreement between phenotypic data and the panel of *bla* genes detected in the strains analysed. The diversity of *bla* genes encountered in isolates from blood, stool and urine specimen of hospitalized patients was almost identical to the panel of genes encountered in corresponding specimens from non-hospitalized patients. This partially suggests a possible exchange of strains between hospitalized and non-hospitalized patients or a flow of genes among strains from different clinical backgrounds. Based on the resistance profiles, we identify ESBL-, CMT- and pAmpC-producers as the most important set of strains whose spread in hospital and community settings should be closely monitored. If the prevalence of isolates with such highly resistant strains continues to rise, majority of β-lactam antibiotics may cease to be effective agents for management of community- and hospital-acquired infections in Kenya.

It is highly likely that heavy use of antibiotics to treat different infections, and urethral tract infections (UTI) in particular, has selected for isolates carrying multiple *bla* genes such as those encountered in this study. Since the antibiotic-use policy is rarely enforced in Kenya, and since most prescriptions are issued without culture and susceptibility data, β-lactam antibiotics are likely to be glossily misused. This may partially explain why complex phenotypes such as ESBL-, CMT- and pAmpC-like phenotypes were observed even among isolates from stool. The current study also shows that 41% of the isolates were resistant to at least one β-lactamase inhibitor. High resistances to inhibitor antibiotics may emerge as a result of over-reliance on amoxicillin-clavulanic acid to treat different infections in Kenya even without a valid prescription. It is however interesting to note that the prevalence of inhibitor resistant *bla* genes is still very low among strains exhibiting an IRT-like phenotype. Similar studies conducted in Spain reported a similar low prevalence of IRTs [29,30]. The only true IRT reported in this study was TEM-103 while majority (75%) of isolates with an IRT-like phenotype carried a combination of *bla*_*TEM*-1_ + *bla*_*OXA*-1_. These two genes were also frequently detected in isolates exhibiting a combination of an ESBL- and CMT-like phenotypes. However, *bla*_*OXA*-1_ and *bla*_*TEM-1*_ were also detected in isolates susceptible to inhibitors. We speculate that besides conferring resistance to narrow spectrum penicillins, some TEM-1 and OXA-1 may be implicated in resistance to other classes of antimicrobials such as various generations of cephalosporins and possibly, β-lactam/β-lactamase inhibitor combinations. These hypothesis is partially based on findings from a recent study conducted in Kenya that described novel *bla*_*OXA*-1_ enzymes in *Salmonella* strains that contain promoter mutations that confer resistance to broad-spectrum β-lactam antibiotics including β-lactamase inhibitors [23]. Furthermore, studies conducted elsewhere have also reported resistance to multiple β-lactam antibiotics due to promoter mutations that result to over-production of TEM-1 enzymes [30]. It is therefore important to further investigate genetic basis of resistance and the role of these otherwise narrow-spectrum β-lactamases (TEM-1 and OXA-1) in mediating resistance to advanced classes of β-lactam antibiotics in developing countries.

In the current study, we found a high diversity of CMTs, yet these enzymes have been reported only in a few countries [13]. It is possible that the ease of access to β-lactam/β-lactamase inhibitor combinations in Kenya without valid susceptibility data has selected for strains with CMT genes that are rarely reported from other countries. In contrast, majority of CTX-M- and SHV-type ESBLs and CMY-type pAmpCs genes identified are those with a global-like spread pattern [31-39]. Similarly, TEM-52, the only TEM-type ESBL reported in this study, is frequently reported in USA [39] and Europe [40]. The wide dissemination of genes encoding these ESBLs and pAmpCs is attributed to physical association between these genes and mobile genetic elements such as ISE*cp*1, transposons and conjugative plasmids [41-43]. Such genetic affiliations further underline the potential of these genes described in this study to spread to susceptible strains through horizontal gene transfer mechanisms.

## Conclusions

This study demonstrates the need to combine phenotypic and molecular methods in order to understand important aspects of resistance to β-lactam antibiotics in developing countries. We recommend that measures be put in place to minimize possible exchange of strains between hospitalized and non-hospitalized patients. Prudent use of β-lactam antibiotics in developing countries should be advocated and in such countries, the existing empiric treatment regimes should be revised occasionally in order to reflect prevailing resistance phenotypes. Such measures may help to preserve the potency of β-lactam antibiotics and improve success of chemotherapy. Finally, the diversity of *bla* genes described in this study is relatively high and majority of genes in circulation among *E. coli* strains investigated have a global-like spread. We recommend that attempts be made to investigate the role of Africa and other developing countries as sources or destinations of β-lactamase-producing strains.

## Methods

### Bacterial strains

Between 1992 and 2010, our laboratory at the KEMRI Centre for Microbiology Research received 912 *E. coli* isolates from 13 health centres in Kenya. All the 912 isolates were resistant to penicillins alone (e.g. ampicillin), or a combination of penicillins and different classes of β-lactam antibiotics. These isolates were from urine (395), blood (202), stool (315) and were obtained from confirmed cases of urethral tract infections (UTIs), septicaemia and diarrhoea-like illnesses respectively. Out of the 912 isolates, 255 (28 %) were obtained between 1992 and 1999 while 657 (72 %) were obtained between 2000 and 2010. This difference was as a result of an increase in isolation rates as a result of better detection and screening techniques in recent years. These isolates were obtained from 350 patients seeking outpatient treatment and 562 were from hospitalised patients. Upon receipt, the isolates were sub-cultured on MacConkey agar (Oxoid, Basingstoke, U`K) and species identification done using standard biochemical tests as described before [44]. Ethical clearance to carry out this study was obtained from the KEMRI/National Ethics Committee (Approval: SSC No. 1177).

### Antimicrobial susceptibility profiles

Antimicrobial susceptibility tests were performed for all the 912 isolates using antibiotic discs (Cypress diagnostics, Langdorp, Belgium) on Mueller Hinton agar (Oxoid, Basingstoke, United Kingdom). *E. coli* ATCC 25922 was included as a control strain on each test occasion. Susceptibility tests were interpreted using the Clinical and Laboratory Standards Institute (CLSI) guidelines [45]. The antibiotics included in this panel were: - ampicillin (AMP, 10 μg), oxacillin (OXA, 30 μg), amoxicillin (AML, 30 μg ), cephalothin (KF, 30 μg), cefuroxime (CXM 30 μg), cefotaxime (CTX, 30 μg) and ceftazidime (CAZ, 30 μg). Other antibiotics included cefepime (FEP, 30 μg), aztreonam (AZT, 30 μg), and cefoxitin (FOX, 30 μg). β-lactam/β-lactamase inhibitor combinations included amoxicillin/clavulanic acid (AMC, comprising amoxicillin 20 μg and clavulanic acid 10 μg), ampicillin/sulbactam (AMS) combinations in rations of 20 μg and 10 μg respectively, and piperacillin/tazobactam (TZP) in potency ratio of 100/10 μg respectively. Imipenem (IM 30 μg) was used to test susceptibility to carbapenems.

### Detection and Interpretation of β-lactamase phenotype

Two strategies were used for detection of β-lactamase phenotypes as detailed in the CLSI guidelines [45], and in other related studies [46]. The first strategy was the double-disc synergy test (m-DDST) in which the β-lactam antibiotics were placed adjacent to the amoxicillin/clavulanic (AMC) disc at inter-disc distances (centre to centre) of 20 mm. A clear extension of the edge of the disc zones towards the AMC (ghost zones or zones of synergy) was interpreted as positive for ESBL production. In the combined disc method (CDM), tests were first done using β-lactam antibiotics and then repeated using discs containing combinations of β-lactam/β-lactamase inhibitors. A result indicating a ≥ 5 mm increase in zone diameter for the β-lactam/β-lactamase inhibitor disc was interpreted as production of ESBLs [45,46]. The results from the m-DDST and CDM methods were also used for empiric categorization of strains into NSBL-, IRT-, ESBL- CMT- and pAmpC-like β-lactamase phenotypes as detailed before [5].

### PCR detection of β-lactamase genes

Preparation of DNA used as template in PCR reactions was obtained by boiling bacteria suspension from an 8 hr culture at 95 °C for 5 minutes. The supernatant was stored at -20^o^ C until further use. Subsequent PCR amplifications were carried out in a final volume of 25 μL or 50 μL. A minimum of 5 μL of template DNA and 1 μL of 10 mM concentration of both forward and reverse primers were used in PCR reactions. Isolates from our collection that had been found to carry various *bla* genes in past studies [24,27,47], were used as positive controls in PCR screening for genes of interest. Sterile distilled water or *E. coli* strains susceptible to all β-lactam antibiotics were used as negative controls. PCR products were analyzed using electrophoresis in 1.5 % agarose gels and stained with ethidium bromide. Visualization of the PCR products was done under UV light and the image recorded with the aid of a gel documentation system (Bio-Rad Laboratories, Hercules, CA, USA).

### Selection of isolates for further analysis

Isolates from each phenotype were selected for further analysis using PCR and sequencing strategies. For phenotypes with a high number of isolates (i.e. more than a hundred strains), at least 56% of the isolates were selected for further analysis. In order to minimize bias, the isolates selected from each phenotype were proportion to the total number of isolates obtained during each year of isolation (1992 to 2010). Similarly, the number of isolates selected from urine, stool and blood specimen was proportional to the total number of strains isolated from each specimen-type obtained from both hospitalized and non-hospitalized patients. Using this criterion, 586 (64%) of the 912 isolates were selected for further analysis. Regardless of the source phenotype, all the selected isolates were investigated for carriage of the complete panel of *bla* genes screened for in this study.

### Screening for *bla* genes

The strains were screened for genes frequently reported among members of family Enterobacteriaceae [11]. The list of primers used is indicated in Table 4. Consensus primers published in past studies were used for screening for *bla*_SHV_ and *bla*_TEM_[48,49], *bla*_CTX-M_[50] and *bla*_CMY_[51]. Isolates positive using *bla*_CTX-M_ consensus primers were screened using primers specific for CTX-M group I to IV as described in a previous study [52]. Isolates positive using the *bla*_CMY_ primers were analyzed using primers for *bla*_CMY-1_-like and *bla*_CMY-2_-like genes [53]. Detection of other β-lactamase genes was done as previously described for *bla*_OXA_-like [53,54], *bla*_PER_-like [55] , *bla*_ACC_-like [53], *bla*_VEB_-like [56], and *bla*_DHA_-like genes [57].

### Sequencing

Amplicons used as template in sequencing reactions were purified using the QIAquick PCR purification kit (Qiagen Ltd., West Sussex, UK). Bi-directional sequencing of the products was done using the DiDeoxy chain termination method in ABI PRISM 310 automatic sequencer (PE Biosystems, Foster City, CA, USA). Consensus primers were used for sequencing except for *bla*_*CTX-M*_ and *bla*_*OXA*_ genes that were sequenced using group-specific primers. Translation of nucleotide sequences was done using bioinformatics tools available at the website of the National Center of Biotechnology Information on http://www.ncbi.nlm.nih.gov. Alignment of the translated enzyme amino acid sequence was done against that of the wild-type using the ClustalW program on http://www.ebi.ac.uk [58]. Identification of enzyme mutations at amino acid level was determined by comparing the translated amino acid sequence with that of the wild-type enzyme published at http://www.lahey.org/studies.

## Competing interests

None of the authors have competing interests.

## Authors’ contributions

JK designed the study, carried out the experiments and wrote the manuscript. SK, BM and PB designed the study and participated in manuscript write-up and review. All authors read and approved the final manuscript.

## Authors’ information

JK and SK are research Scientist at the Kenya Medical Research Institute (KEMRI). BMG is Professor at the K.U.Leuven (Faculty of Bioscience Engineering) while PB is a Senior Research Scientist at the Veterinary and Agrochemical Research Centre (VAR).
